# Study on semi-bionic extraction of Astragalus polysaccharide and its anti-aging activity *in vivo*

**DOI:** 10.3389/fnut.2023.1201919

**Published:** 2023-07-17

**Authors:** Xinlei Yan, Jing Miao, Bao Zhang, Huan Liu, Huifang Ma, Yufei Sun, Pufang Liu, Xiujuan Zhang, Ruigang Wang, Juntao Kan, Feiyun Yang, Qiming Wu

**Affiliations:** ^1^College of Food Science and Engineering, Inner Mongolia Agricultural University, Hohhot, China; ^2^The Institute of Biotechnology, Inner Mongolia Academy of Science and Technology, Hohhot, China; ^3^College of Life Sciences, Inner Mongolia Key Laboratory of Plants Adversity Adaptation and Genetic Improvement in Cold and Arid Regions of Inner Mongolia, Inner Mongolia Agricultural University, Hohhot, China; ^4^Nutrilite Health Institute, Shanghai, China

**Keywords:** Astragalus polysaccharide, separation, purification, antioxidant, anti-aging, D-galactose

## Abstract

*Astragalus membranaceus* (*A. membranaceus*) is a homologous plant with high medicinal and edible value. Therefore, the extraction methods of Astragalus polysaccharide (APS) have attracted the attention of many research groups, but the yield of the active components is still not high. The aim of this study was to extract APS by a semi-bionic extraction method, optimize the extraction process, and evaluate the anti-aging activities of APS *in vivo*. The results showed that the APS yield was 18.23% when extracted by the semi-bionic extraction method. Anti-aging evaluation in rats showed that APS extracted by this method significantly decreased the malondialdehyde (MDA) content and increased superoxide dismutase (SOD) activity to cope with D-galactose-induced aging. Serum metabolomic analysis indicated that a total of 48 potential biomarkers showed significant differences, mainly involving 5 metabolic pathways. These altered metabolic pathways were mainly related to energy metabolism, amino acid metabolism, and lipid metabolism. These results indicated that the semi-bionic extraction method can effectively improve the yield of APS, and the extracted APS exhibited anti-aging activity in rats. Our study provided a novel and effective method to extract APS and indicated that APS can be used as functional food and natural medicine to delay aging and prevent its complications.

## 1. Introduction

*Astragalus membranaceus* (*A. membranaceus*) belongs to the Leguminosae family. The root of *A. membranaceus* is widely used in the food and pharmaceutical industries. Previous studies have shown that *A. membranaceus* has anti-inflammatory, anti-swelling, antitumor and antioxidant activities (1–3). This material was listed as a drug-food homologous substance in 2018, greatly enriching the market of new functional foods. Studies have shown that one of the main effective components of *A. membranaceus* is Astragalus polysaccharide (APS), which have the effects of cardiovascular protection, immune function regulation, antitumor activity, and metabolism improvement (4–6). APS can effectively exert antioxidant and anti-aging effects and reduce blood glucose levels (7). Moreover, APS is an active ingredient of great significance for the development of new functional foods.

To date, the commonly used methods for polysaccharide extraction include water extraction, microwave extraction and enzymatic extraction. Water extraction involves soaking raw materials in hot water or at room temperature to extract polysaccharides. This method has simple operation and low cost, but in the extraction process, the extraction temperature is usually high, the structure of polysaccharide is easy to be destroyed, and the other disadvantages of this method are long extraction time and low extraction efficiency (8). Microwave-assisted extraction technology uses microwave to increase the pressure inside the cells of biological materials, rupture the cell wall, and immediately dissolve the active components in the cells, but this method is easy to destroy the structure of polysaccharide and has a low extraction yield (Tang et al., 2022). The principle of enzymatic extraction of polysaccharides lies in the specific catalytic action of enzymes. The enzyme extraction conditions are mild and the extraction yield is high, but the actual production cost is high (9). Therefore, it is necessary to seek a high extraction rate, no damage to the structure of polysaccharide, cost economic extraction method. The semi-bionic extraction method, a new technology for extracting medicinal materials, was first proposed by Wang (10). The principle of this method is to simulate oral administration and drug transport through the gastrointestinal tract, which is different from the acid–base method. It not only complies with the guidelines for the use of traditional Chinese medicine but also improves the efficacy and does not damage its structure. Therefore, this study aimed to optimize the APS extraction process and obtain an economical and convenient method that can improve the yield of polysaccharides.

APS plays a prominent role in regulating the body’s immune function, scavenging free radicals and protecting against lipid peroxidation (11–13). Liu et al. showed that APS could improve the symptoms of type II diabetic rats and regulate insulin signaling (14). It was reported that APS could also be used as a therapeutic agent to inhibit muscle atrophy by restoring the phosphorylation of Akt, m-TOR, and P70s6k (15). Previous studies have reported the anti-aging activity of APS *in vitro*, but there are few studies on the anti-aging activity and mechanism of APS *in vivo*. In this study, the anti-aging degree of rats was evaluated by the content of superoxide dismutase (SOD) and malondialdehyde (MDA) (16, 17). Metabolomic analysis and identification of metabolites provide ideas for the study of the anti-aging mechanism of APS and provide a certain theoretical basis for the development of functional foods and natural drugs in the future.

## 2. Materials and methods

### 2.1. Extraction of APS

Dried *A. membranaceus* powder was provided by Zhang Xiujuan’s team of Inner Mongolia Academy of Science and Technology. A certain amount of dried *A. membranaceus* powder was added to 10 times the volume of simulated gastric juice (2.0 g sodium chloride and 7 mL hydrochloric acid were filled with distilled water to 1,000 mL, and the pH was adjusted to 1.5 ± 0.1) and extracted at 100°C for 1 h. After centrifugation (4,000 r/min, 10 min), the supernatant was collected and filtered to obtain the simulated gastric juice extract. The precipitate was dissolved in 10 times the volume of simulated intestinal fluid (7 g potassium dihydrogen phosphate was dissolved in 250 mL distilled water, then 70 mL 0.2 mol/L sodium hydroxide solution and 500 mL distilled water were added, and the distilled water was added to 1,000 mL. The pH was adjusted to 7.0 ± 0.1) and extracted at 100°C for 1 h. After centrifugation, the supernatant was collected and filtered to obtain the simulated intestinal fluid extract. Simulated gastric juice and simulated intestinal juice extracts were combined, ethanol was added, overnight, and crude APS were obtained by centrifugation. In the single-factor experiment, the ratio of *A. membranaceus* powder to liquid was 1:10, 1:15, 1:20, 1:25 and 1:30; extraction times were 30 min, 60 min, 90 min, 120 min, and 150 min; extraction temperatures were 40°C, 55°C, 70°C, 85°C, and 100°C. On the basis of single-factor experiments, orthogonal experiments were carried out to obtain the best extraction process, and the level settings of each factor were shown in Supplementary Table S1.

### 2.2. Determination of total sugar content of APS

The total sugar content of APS was determined by phenol-sulfuric acid method. The solution of 0.5 mg/mL APS was prepared and 30 uL was added into the 96-well plate. Then 30 uL 5% phenol solution and 150 uL concentrated sulfuric acid were added and mixed. After 30 min at room temperature, the absorption value was measured at 490 nm. The total sugar content of APS was calculated according to glucose standard curve. The total sugar content of polysaccharides is the measured polysaccharide mass (g) divided by the weighed polysaccharide mass (g).

### 2.3. Purification of APS

The protein of extracted crude APS was removed by using of Sevage reagent(the volume ratio of chloroform and n-butanol was 4:1). 2 mg/mL crude APS solution was prepared and 50 mL was used for extraction, then 200 mL of Sevage reagent was added, and the solution was shaken at 180 rpm for 30 min. The supernatant was centrifuged at 4,000 rpm for 10 min, repeated 4 times, and the precipitate was discarded to obtain APS. Dialysis was then performed with a 7 kDa dialysis bag (Yeasen Biotechnology Co., Ltd., Shanghai). After dialysis, the pigment was removed by magnetic stirring of s-8 macroporous resin, the ratio of polysaccharide to macroporous resin was 1 mL: 10 g, and the APS solution was obtained by magnetic stirring at 40°C for 60 min and the filtered by filter paper. Finally, APS was subjected to column chromatography using a DEAE-52 cellulose chromatographic column (Ф2.5 × 50 cm) (Ruidahenghui Biotechnology Co., Ltd., Beijing) with 200 mL deionized water and 0.1 mmol/L NaCl solution for polysaccharide elution at room temperature, with an average flow rate of 1 mL/min, and 40 tubes of eluate were collected every 10 mL. The absorbance value was measured at 490 nm by the phenol-sulfuric acid method, and the elution curve was drawn. Finally, eluates with absorbance values higher than 0.5 were combined and freeze-dried to obtain astragalus polysaccharides (18–20).

### 2.4. Morphological observation of Astragalus polysaccharides

The dried APS powder was adhesive to the conductive glue, and the excess powder was blown away by nitrogen gas. The film was sprayed with gold by JFC-1600 ion sputter, and observed by Apreo S LoVac scanning electron microscope at different times (2,400, 10,000).

### 2.5. Animals and experimental design

6-week-old female SD rats (body weight of 150 to 180 g) were purchased from SPF (Beijing) Biotechnology Co., Ltd., Beijing, China. All mice were fed with *ad libitum* food and water for free, and the mice were housed under a 12 h light/dark cycle. All animal research was approved by the Inner Mongolia Agricultural University Animal Experiment Ethics Inspection Committee (approval No.: NND20210069) and complied with the guidelines of the Laboratory Animal Welfare and Ethics of Inner Mongolia Agricultural University.

After adaptive feeding for 2 weeks, the rats were randomly divided into four groups (blank, model, low-dose and high-dose), with 7 rats in each group, and the total sugar content of the APS used in this experiment was 62.36%. The blank group was intraperitoneally injected with 5 mL/kg normal saline per day, and the other 3 groups were intraperitoneally injected with 120 mg/kg D-galactose per day to establish an aging model. The rats in the low-dose group were treated with 100 mg/kg per day by gavage, those in the high-dose group were treated with APS at 400 mg/kg per day, and those in the blank group and the model group were treated with the same volume of normal saline every day for 8 weeks. After gavage for the last time, the animals in each group were fasted for 12 h and anaesthetized with 10% chloral hydrate. Blood was taken from the abdominal aorta and centrifuged at 4°C and 3,500 r/min for 15 min, and the supernatant was collected, packed and refrigerated at −80°C (21).

### 2.6. Detection of serum SOD and MDA

The rat serum was centrifuged at 2,000 rpm for 20 min, and the supernatant was collected and prepared. The concentration of SOD and MDA was measured using the commercial kit (Jiancheng Tech, Nanjing), according to the instruction supplied by manufacturer.

### 2.7. Detection of metabolites in the serum samples of the anti-aging rats

The serum samples were pretreated and used for mass spectrometry analysis, PCR analysis and PLS-DA. Then, ions with VIP > 1 and *p* < 0.05 were selected as potential characteristic biomarkers. Potential biomarkers were then matched to the Human Metabolome Database (HMDB)^1^ and mzCloud^2^ with information. Finally, Metabo Analyst^3^ analysis was used to identify metabolic pathways and their significance (22).

### 2.8. Statistical analysis

All experiments were performed in triplicate, and the results are presented as the means ± standard deviations (SD). SPSS 21.0 software (SPSS Inc., Chicago, IL, United States) was used to conduct one-way analysis of variance (ANOVA). *p* < 0.05 or *p* < 0.01 was considered statistically significant.

## 3. Results

### 3.1. Extraction of APS

A single-factor experimental study was conducted on the semi-bionic extraction method, and the yield of APS was used as the evaluation basis. The single-factor experimental results showed (Table 1) that the crude polysaccharide yield was 16.23%. The orthogonal experimental design and results showed that (Tables 2, 3) the experimental Group A_1_B_3_C_2_ (a ratio of solid to liquid of 1:10, an extraction temperature of 100°C, and an extraction time of 60 min) was the best extraction condition of APS in this study.

**Table 1:** Single-factor experimental results of the semi-bionic extraction method.

**Factor**	**Material-liquid ratio**	**Yield of Astragalus Polysaccharide/%**	**Extraction temperature/°C**	**Yield of Astragalus Polysaccharide/%**	**Extraction Time/min**	**Yield of Astragalus Polysaccharide/%**
1	1:10	16.21 ± 0.78^a^	40	5.23 ± 1.45^d^	30	9.65 ± 1.20^b^
2	1:15	15.35 ± 1.24^a^	55	6.31 ± 1.11^d^	60	16.10 ± 0.69^a^
3	1:20	12.97 ± 2.12^b^	70	9.62 ± 0.73^c^	90	15.83 ± 1.24^a^
4	1:25	11.12 ± 2.41^b^	85	13.04 ± 1.01^b^	120	16.31 ± 1.77^a^
5	1:30	10.23 ± 1.55^b^	100	16.11 ± 0.59^a^	150	16.39 ± 0.60^a^

The results were displayed as the mean ± SD. Different lowercase letters a, b, c and d indicate significant differences at the *p* < 0.05 level.

**Table 2:** Results of the semi-bionic extraction orthogonal experiment.

Number of experimental group	Solid–liquid ratio	Extraction time/Min	Extraction temperature/°C	Yield of Astragalus Polysaccharide/%
1	1:10	70	30	8.32
2	1:10	85	60	11.84
3	1:10	100	90	16.41
4	1:15	70	60	9.53
5	1:15	85	90	12.42
6	1:15	100	30	10.51
7	1:20	70	90	6.83
8	1:20	85	30	7.72
9	1:20	100	60	15.21
Mean value1	12.167	8.200	8.833	
Mean value2	10.800	10.633	12.167	
Mean value3	9.900	14.033	11.867	
Range	2.267	5.833	3.334	
Level of excellence	A_1_	B_3_	C_2_	

According to the range value of each influencing factor, the order of influence on the extraction rate of APS was extraction temperature > extraction time > solid–liquid ratio; that is, the extraction temperature had the greatest influence on the extraction rate of APS. According to the range value, the optimal level of orthogonal experiments is A_1_B_3_C_2_.

**Table 3:** Analysis of variance of each factor of the orthogonal experiment.

Factor	Sum of squares of deviation	degree of freedom	*F*-ratio	*F* critical value
Solid–liquid ratio	7.816	2	0.294	5.140
Extraction temperature	51.509	2	1.938	5.140
Extraction time	20.102	2	0.768	5.140
Error	79.73	6		

### 3.2. Isolation and purification of APS

**Figure 1:**
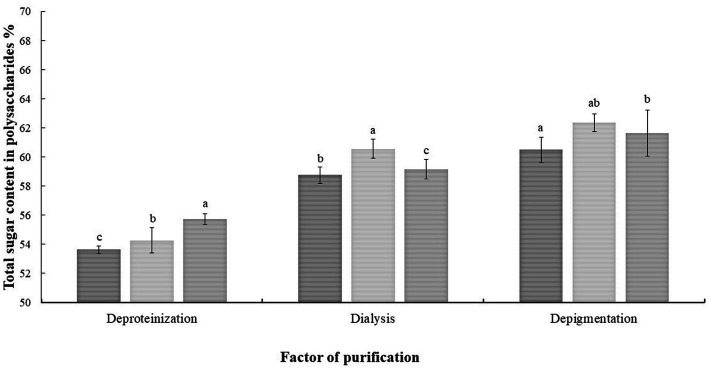
Analysis of variance of each factor of the orthogonal experiment. The results were displayed as the mean ± SD. Different lowercase letters a, b and c indicate significant differences at the *p* < 0.05 level.

The purification conditions were shown in Figure 1. The total sugar content was 55.73% after removing protein by the Sevage method. The polysaccharide content was 60.57% after dialysis using a 7 kDa pore diameter dialysis bag. The polysaccharide content was 62.36% after disposing of S-8 macroporous resin.

### 3.3. Scanning electron microscopy analysis

**Figure 2:**
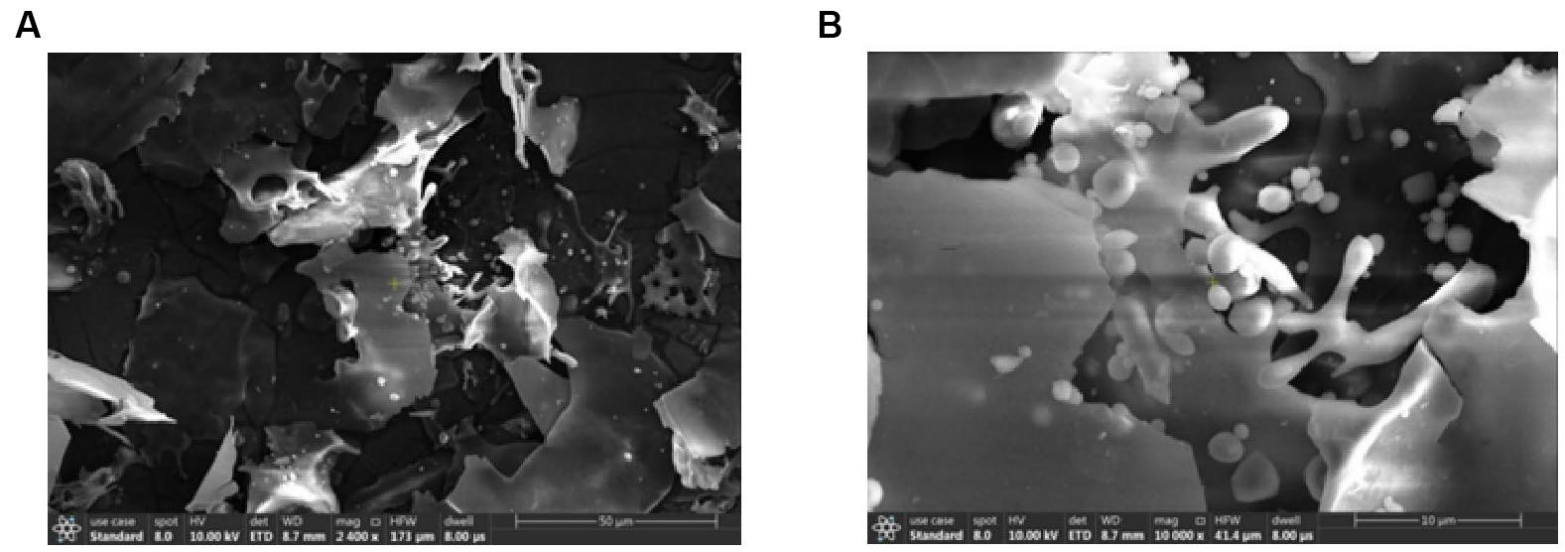
Scanning electron microscopy results of the purified APS. Panel **(A)** showed the results under a 2,400× magnification microscope, and the purified APS were stacked irregularly in sheets or fragments. Panel **(B)** showed the results at 10,000× magnification; the polysaccharide exhibits bulk and rod-like structures with the presence of spherical particles.

The scanning electron microscopy results of the purified APS were shown in Figure 2. As shown in Figure 2A, the purified APS was irregularly sheet-like or fragmented, with a smooth surface, indicating that there was an interaction between polysaccharide molecules, which could form a compact structure. Figure 2B showed the results at 10,000x magnification, where both bulk and rod-like structures can be observed in the presence of spherical particles. According to the literature reports, acidic polysaccharides have particles present in the microstructure. By comparison, it was found that its structure was similar to that of honey-processed APS, showing irregular sheets and obvious spherical granule protrusion.

### 3.4. Study on the anti-aging activity of APS

According to the experimental results (Table 4), compared with the blank group, the SOD activity of the model group was significantly decreased (*p* < 0.01), indicating that the blood SOD activity of the rats induced by D-galactose decreased, which was an important indicator of aging symptoms. Compared with the model group, the activities of SOD in the low-dose and high-dose APS groups were significantly increased (*p* < 0.05), indicating that APS intervention can significantly improve the antioxidant capacity of the blood in aging rats.

**Table 4:** Effect of APS on SOD and MDA activity.

Group	SOD (U/mL)	MDA (nmol/mL)
Blank	728.73 ± 55.40	7.60 ± 1.48
Model	536.58 ± 29.10**	12.14 ± 2.23**
Low dose	621.96 ± 118.05^#^	9.12 ± 1.83^#^
High dose	697.51 ± 61.51^##^	8.85 ± 2.13^#^

Results are displayed as the mean ± SD. Compared with the blank group, *p < 0.05, **p < 0.01; Compared with the model group, #*p* < 0.05, ##*p* < 0.01.

The MDA content in the serum of the aging model group was significantly higher than that of the blank group (*p* < 0.01). Compared with that of rats in the model group, the MDA content of rats in the low-dose and high-dose APS groups decreased. The MDA content of the low- and high-dose groups was significantly lower than that of the aging model group (*p* < 0.05), indicating that APS could reduce the formation of lipid peroxidation products in the blood of aging rats.

### 3.5. Multivariate analysis of serum differential metabolites

**Figure 3:**
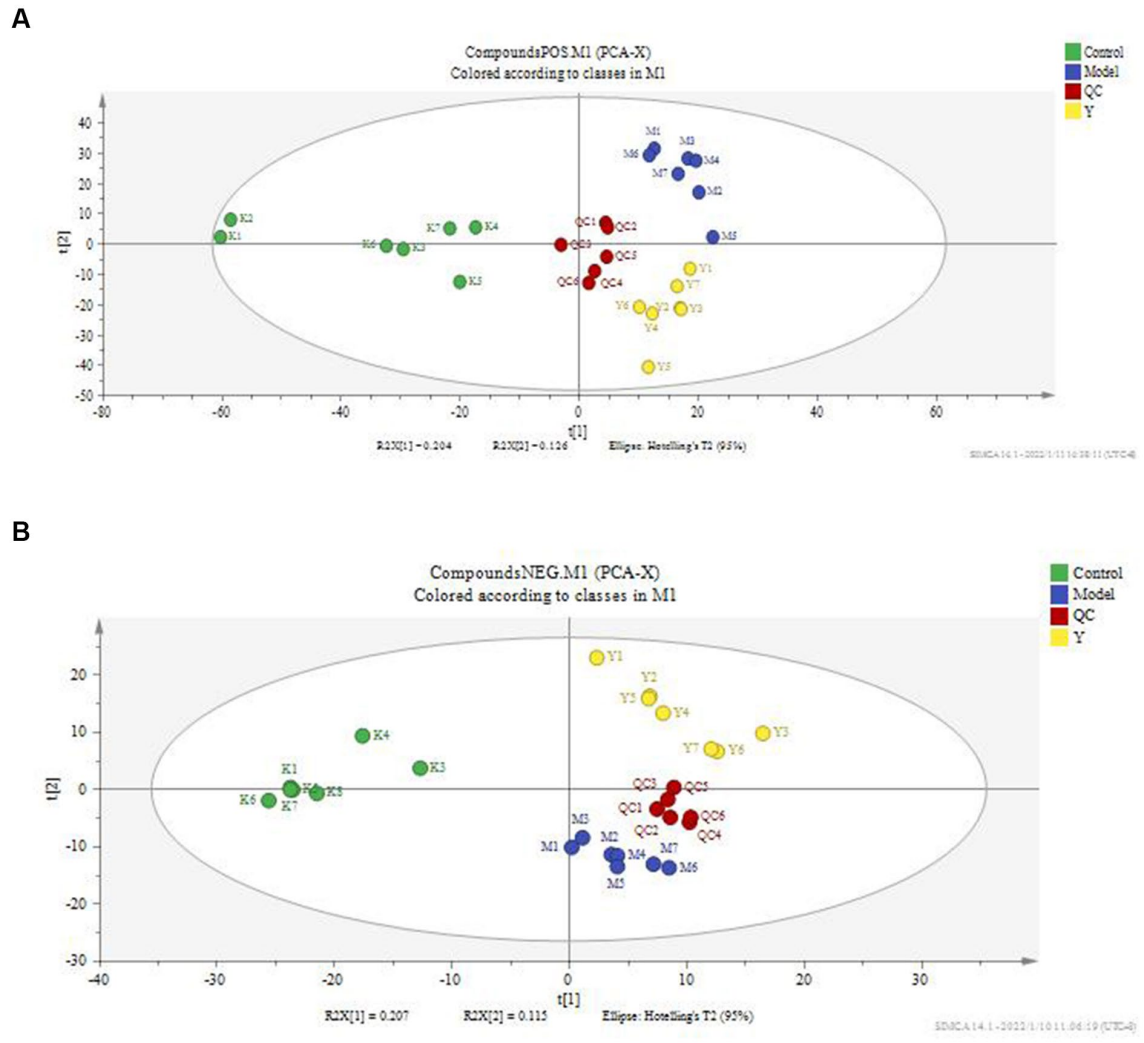
PCA score plot of rat serum. PCA was performed using SIMCA-P 14.1 for rat serum metabolites. **(A)** Positive ion mode; **(B)** negative ion mode; Control: control group; Model: set of models; QC: quality control sample; Y: Astragalus polysaccharide high-dose group.

**Figure 4:**
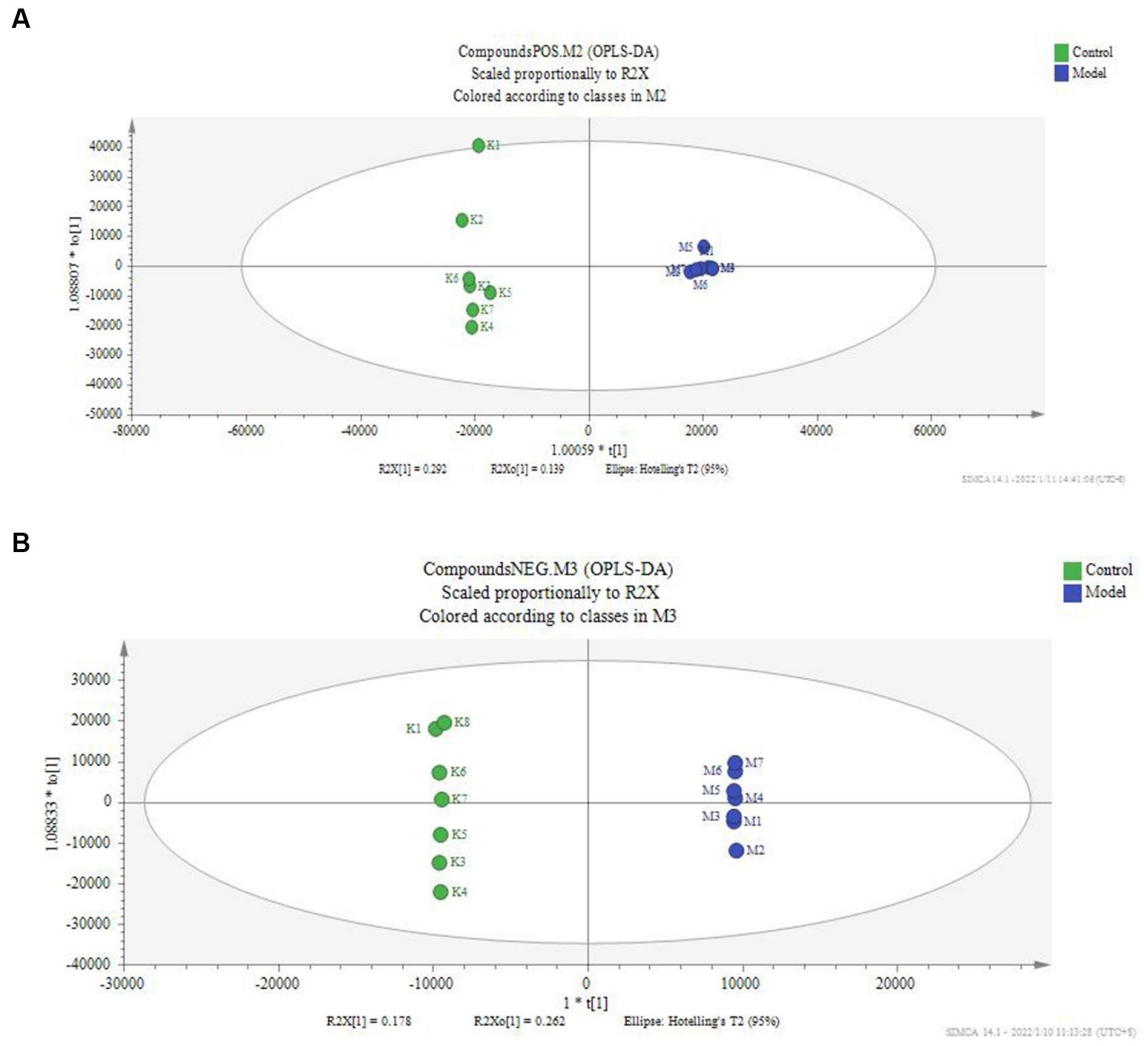
OPLS-DA score of serum of the model group and control group. The OPLS-DA model was used to compare the serum metabolites of different groups, and the OPLS-DA score map of serum metabolites of the model group and the blank group was calculated. **(A)** Positive ion mode; **(B)** negative ion mode; Control: control group; Model: set of models.

**Figure 5:**
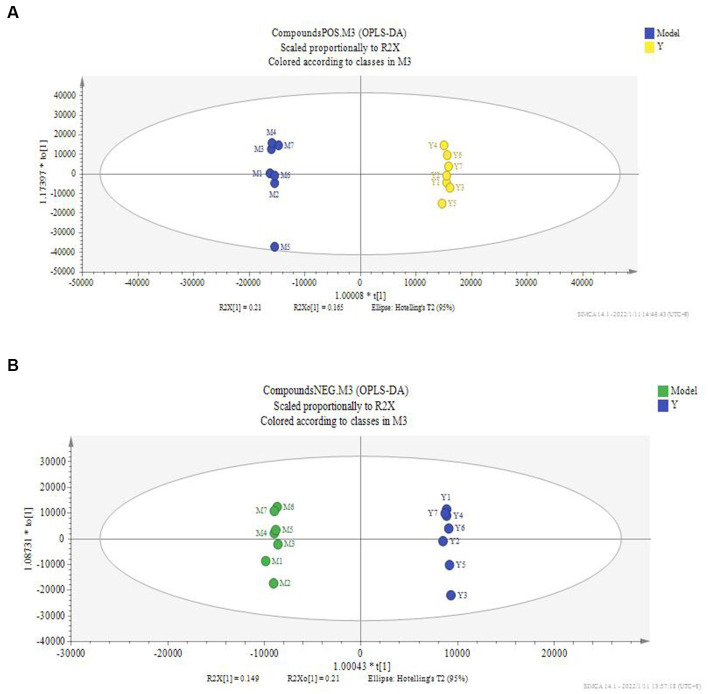
OPLS-DA score of serum of administration group and model group. The OPLS-DA model was used to compare the serum metabolites of different groups, and the OPLS-DA score plots of serum metabolites of rats in the administration and model groups. **(A)** Positive ion mode; **(B)** negative ion mode; Model: set of models; Y: Astragalus polysaccharide high-dose group.

According to the serum metabolite diagram (Figure 3) and OPLS-DA model diagram (Figures 4, 5), the high-dose APS administration group was significantly separated from the model group and tended to the control group, indicating that high-dose APS administration had a certain regulatory effect on the aging rats induced by D-gal.

**Figure 6:**
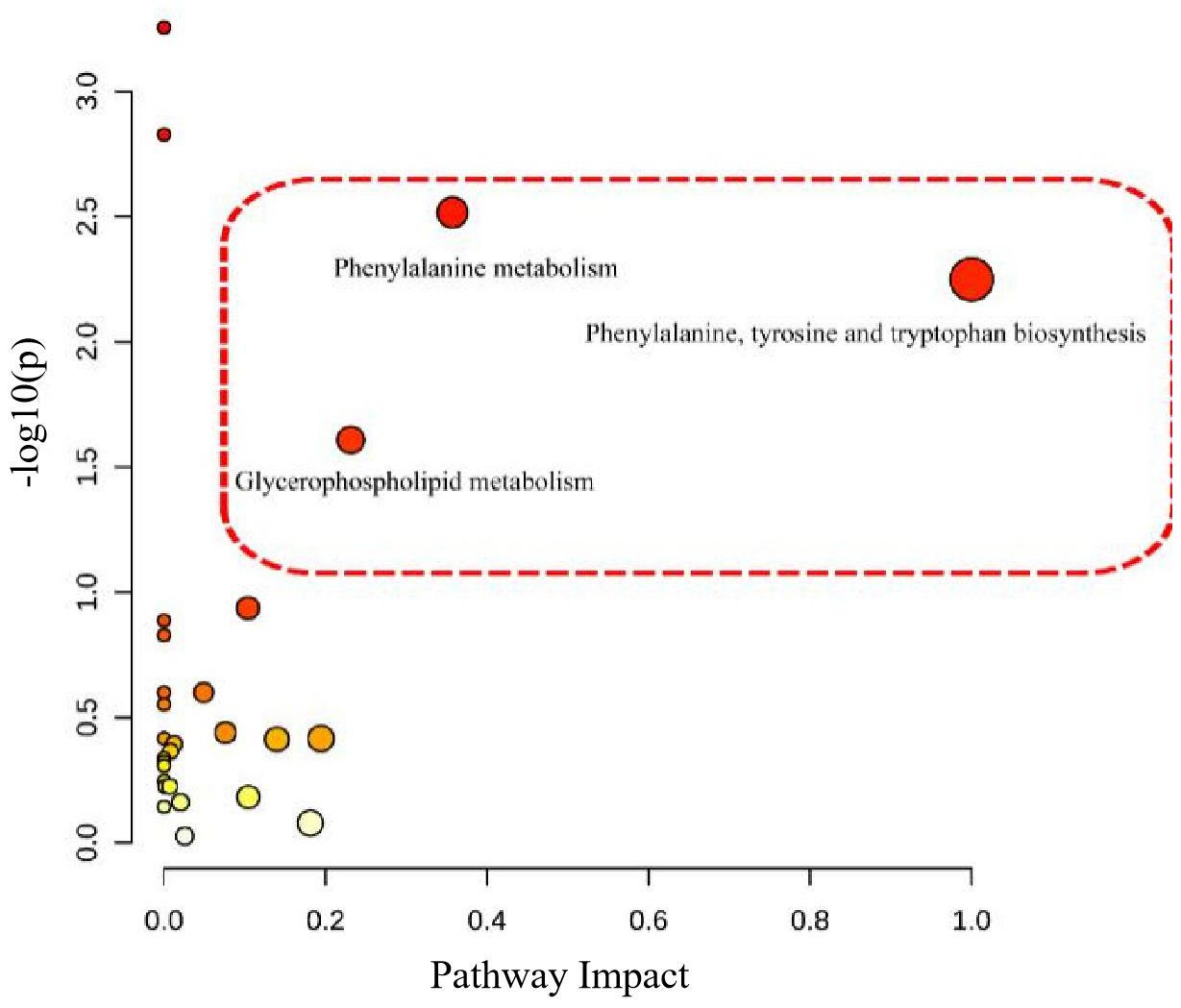
Differential metabolic pathway analysis of model group and control group. The relevant thresholds were set as follows: −log (*P*) > 1.0 and pathway impact value >0.2. The ordinate represents −log (*P*), and the darker the color, the higher the significance level. The horizontal axis represents the importance of metabolic pathways, and the larger the circle, the more important the pathway. Three metabolic pathways were interfered, namely, phenylalanine metabolism; Biosynthesis of phenylalanine, tyrosine and tryptophan; Glycerol phospholipid metabolism.

**Figure 7:**
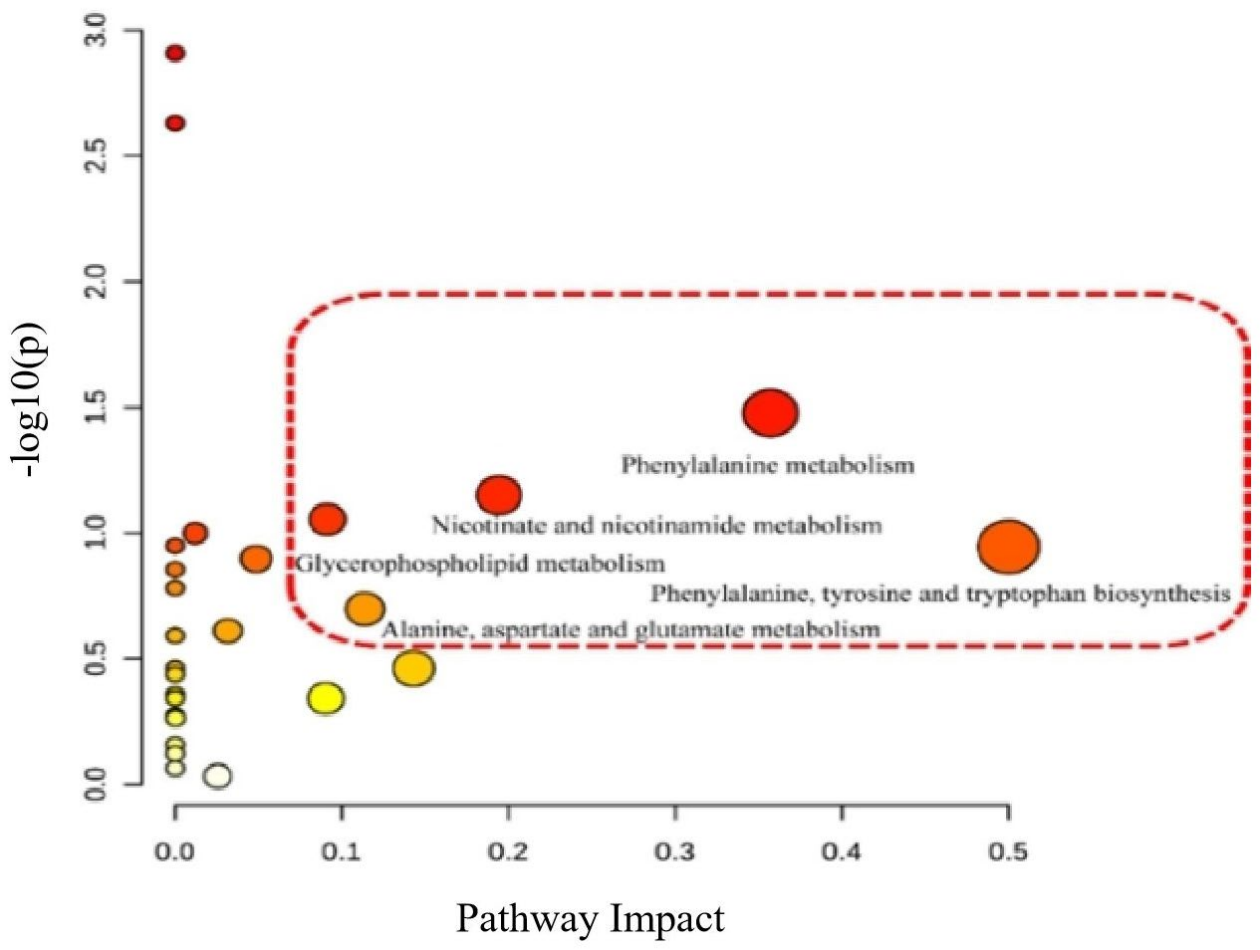
Differential metabolic pathway analysis of model group and administration group. The relevant thresholds were set as follows: −log (*P*) > 1.0 and pathway impact value >0.2. The ordinate represents −log (*P*), and the darker the color, the higher the significance level. The horizontal axis represents the importance of metabolic pathways, and the larger the circle, the more important the pathway. Five metabolic pathways were interfered: phenylalanine metabolism; Biosynthesis of phenylalanine, tyrosine and tryptophan; Niacin and niacinamide metabolism; Glycerol phospholipid metabolism; Alanine, aspartic acid, and glutamic acid metabolism.

According to the above results, the metabolic characteristics of APS-treated rats were different from those of normal rats. Based on the potential biomarkers identified (Supplementary Tables S2, S3), pathway enrichment analysis was performed using the Metabo Analyst database. Five metabolic pathways were affected by APS treatment (Figures 6, 7), including phenylalanine metabolism; biosynthesis of phenylalanine, tyrosine and tryptophan; niacin and nicotinamide metabolism; glycerophospholipid metabolism; and alanine, aspartate, and glutamate metabolism, which had the greatest effects.

## 4. Discussion

The semi-bionic extraction method is a special extraction technology that simulates the human gastrointestinal environment and conforms to the guidelines for the use of traditional Chinese medicine. It has the characteristics of economy, convenience, no residue and no damage to the active structure. However, there have been few studies on the semi-bionic extraction of APS. In this study, the semi-bionic extraction method was used to separate and extract APS. The results showed that the extraction rate of polysaccharides by this method was significantly higher than that of other methods, and the extraction rate reached 18.38%. Li et al. compared the traditional water extraction method and the semi-bionic extraction method for extracting Cassia Poria polysaccharide, showing that the total polysaccharide content of the semi-bionic extraction method was increased by 2.98% compared with the traditional water extraction method (23). Hao et al. compared aqueous extraction and semi-bionic extraction of Auricularia polysaccharide, Corn whisker polysaccharide and Schisandra polysaccharide, and the results showed that the extraction rate of the semi-bionic extraction method was significantly higher than that of traditional water extraction and alcohol precipitation methods on different raw materials (24–26). Therefore, semi-bionic extraction technology provides an idea for the extraction and application of polysaccharides.

Although the anti-aging effect of APS has been reported, most of them have only studied the anti-aging effect of APS *in vitro*. Studies have shown that APS has scavenging ability of DPPH free radicals and hydroxyl free radicals and reducing ability on iron ions, thus proving that APS has an anti-aging effect *in vitro* (27). However, the anti-aging effect and mechanism *in vivo* are still uncertain. In this study, the anti-aging activity of APS was studied in a D-gal-induced aging rat model. The results showed that APS could significantly increase the level of SOD in the diseased rats and significantly reduce the expression of MDA in the diseased rats, indicating that APS affected the metabolism and reduced the oxidative damage of the diseased rats.

Through metabolomics studies, the anti-aging pathways of APS were attributed to amino acid metabolism pathways. Changes in metabolic gene expression and levels of important amino acids are characteristics associated with aging animals (28, 29). In this study, two different amino acid metabolic pathways were identified by pathway enrichment analysis of five pathways. One pathway is that of phenylalanine, tyrosine, and tryptophan metabolism. Phenylalanine is an essential amino acid for the human body and needs to be obtained from food. With increasing age, the synthesis rate of proteins and hormones slows down, the metabolic function of the body is disordered, and the metabolism of phenylalanine in the body changes significantly (30, 31). Tyrosine is derived from phenylalanine and is involved in energy metabolism and scavenging free radicals (32). Tyrosinase catalyzes the conversion of tyrosine to melanin, which is a powerful cation chelator that can absorb free radicals (33). Tyrosine can also be converted into fumaric acid and acetoacetate to participate in the tricarboxylic acid cycle and provide energy for various biochemical reactions, such as oxidative stress regulation (34). APS can change the content of phenylalanine in aging rats, which may be related to its ability to delay aging. The other metabolic pathway is alanine, aspartate, and glutamate metabolism. Alanine itself has no antioxidant effect, but it can synthesize carnosine *in vivo*. Carnosine has a significant scavenging effect on DPPH free radicals and has a positive effect on anti-skin aging (35). In addition, some studies have found that supplementation with alanine can enhance the antioxidant capacity of mice (36). Aspartic acid may also have antioxidant properties, and soaking in aspartic acid solution can effectively inhibit the oxidative browning of fresh-cut potato slices (37).

## 5. Conclusion

The semi-bionic extraction method was used in this study, and the results showed that the method could improve the yield of polysaccharides. In addition, anti-aging experiments showed that APS has the functions of delaying aging, scavenging free radicals, alleviating oxidative stress and improving the metabolism of amino acids and lipids in the body, which is helpful to strengthen our understanding of the biological activity of functional foods. This study provided new insights into the interaction between functional foods and amino acid metabolism. However, the precise relationship between these potential biomarkers and signaling pathways remains limited and needs to be clearly elucidated in further studies.

## Data availability statement

The original contributions presented in the study are included in the article/Supplementary material, further inquiries can be directed to the corresponding authors.

## Ethics statement

The animal study was reviewed and approved by Laboratory Animal Welfare and Animal Experiment Ethics Inspection Committee of Inner Mongolia Agricultural University (approval No.: NND20210069).

## Author contributions

XY and JM drafted the manuscript. BZ, JM, HM, HL, and PL performed the experiments and analyzed the data obtained. RW devised the main conceptual ideas and supervised the experiments. YS and JK revised the manuscript. XZ provided the materials. FY and QW designed the study and analyzed the results. All authors contributed to prepare and review the manuscript.

## Funding

This research was funded by the Science and Technology Project of Inner Mongolia, grant number 2021GG0365; and Project of Introduction of High-level Personnel of Inner Mongolia Agricultural University, grant number NDYB2019-34.

## Conflict of interest

The authors declare that the research was conducted in the absence of any commercial or financial relationships that could be construed as a potential conflict of interest.

## Publisher’s note

All claims expressed in this article are solely those of the authors and do not necessarily represent those of their affiliated organizations, or those of the publisher, the editors and the reviewers. Any product that may be evaluated in this article, or claim that may be made by its manufacturer, is not guaranteed or endorsed by the publisher.

## Footnotes

^1^http://www.hmdb.ca/

^2^https://www.mzcloud.org/

^3^http://www.metaboanalyst.ca/
