# Supplementary material for: Study on semi-bionic extraction of Astragalus polysaccharide and its anti-aging activity in vivo
Source: Front Nutr. 2023 Jul 17;10:1201919. doi: 10.3389/fnut.2023.1201919 (PMC10389262; doi:10.3389/fnut.2023.1201919)
Supplement: Supplementary file 1 [file Table_1.docx]

**Table S1.** Factor level of the orthogonal experiment

| **Factor** | **A Solid-liquid Ratio** | **B Extraction Time**  **/Min** | **C Extraction Temperature**  **/℃** |
| --- | --- | --- | --- |
| 1 | 1:10 | 30 | 70 |
| 2 | 1:15 | 60 | 85 |
| 3 | 1:20 | 90 | 100 |

On the basis of the single-factor experiment, the optimal region of each factor was selected, and three factors with a relatively large influence on the solid‒liquid ratio, extraction time and extraction temperature were selected for orthogonal experiment.
