# Supplementary material for: Study on semi-bionic extraction of Astragalus polysaccharide and its anti-aging activity in vivo
Source: Front Nutr. 2023 Jul 17;10:1201919. doi: 10.3389/fnut.2023.1201919 (PMC10389262; doi:10.3389/fnut.2023.1201919)
Supplement: Supplementary file 2 [file Table_2.docx]

**Table S2.** Differential metabolites in rat serum in the model group and control group

| **Number** | **metabolite** | **molecular formula** | **Parent ion** | **m/z** | | **Deviation**  **/ppm** | **retention time/min** | | **VIP** |
| --- | --- | --- | --- | --- | --- | --- | --- | --- | --- |
|  |  |  |  | **measured value** | **predicted value** |  |  |  |  |
| 1 | L-phenylalanine | C9H11NO2 | [M+H]+ | 165.079 | 165.079 | 0.25 | 0.84 | | 5.61 |
| 2 | Choline palmitoyl glycerophosphate | C24H50NO7P | [M+H]+ | 495.333 | 495.332 | 0.97 | 18.14 | | 4.75 |
| 3 | Acetyl L-carnitine | C9H17NO4 | [M+H]+ | 203.116 | 203.116 | -1.12 | 0.73 | | 4.60 |
| 4 | valine | C5H11NO2 | [M+H]+ | 117.079 | 117.079 | 1.81 | 0.74 | | 4.49 |
| 5 | D-tryptophan | C11H12N2O2 | [M+H]+ | 204.090 | 204.090 | 0.40 | 3.19 | | 4.33 |
| 6 | 1-oleoyl glycerophosphate choline | C26H50NO7P | [M+H]+ | 519.333 | 519.333 | 1.21 | 17.88 | | 4.32 |
| 7 | Indole acrylic acid | C11H9NO2 | [M+H]+ | 187.064 | 187.063 | 1.18 | 3.14 | | 4.25 |
| 8 | Leucine | C6H13NO2 | [M+H]+ | 131.095 | 131.095 | 0.93 | 0.97 | | 4.12 |
| 9 | 1-oleoyl glycerophosphocholine | C26H52NO7P | [M+H]+ | 521.349 | 521.348 | 1.25 | 18.29 | | 4.09 |
| 10 | Nicotinamide | C6H6N2O | [M+H]+ | 122.048 | 122.048 | 1.86 | 0.77 | | 3.97 |
| 11 | N-acetyl-l-o leucine | C8H15NO3 | [M+H]+ | 173.105 | 173.105 | 0.90 | 1.05 | | 3.73 |
| 12 | L-leucine | C6H13NO2 | [M+H]+ | 131.095 | 131.095 | 0.62 | 1.21 | | 3.40 |
| 13 | Bile acid | C24H40O5 | [M-H]- | 408.287 | 408.288 | -0.99 | 15.66 | | 3.21 |
| 14 | creatine | C4H9N3O2 | [M+H]+ | 131.070 | 131.070 | 2.24 | 0.74 | | 3.08 |
| 15 | Monoethylhexyl phthalic acid | C16H22O4 | [M+H]+ | 278.152 | 278.152 | 0.07 | 11.93 | | 3.06 |
| 16 | isoleucine | C6H13NO2 | [M+H]+ | 131.094 | 131.095 | -3.88 | 0.85 | | 3.03 |
| 17 | L-subglycine | C7H11NO2 | [M+H]+ | 141.079 | 141.079 | 1.50 | 0.78 | | 2.79 |
| 18 | 8-amino-7-oxazononate | C9H17NO3 | [M+H]+ | 187.121 | 187.121 | -0.61 | 0.81 | | 2.59 |
| 19 | 1-hydroxy-2-naphthoate | C11H8O3 | [M-H]- | 188.048 | 188.047 | 3.97 | 0.84 | | 2.59 |
| 20 | Dibutyl phthalate | C16H22O4 | [M+H]+ | 278.152 | 278.152 | 0.18 | 15.65 | | 2.50 |
| 21 | Hydroxyproline-leucine | C11H20N2O3 | [M+H]+ | 228.147 | 228.147 | -0.89 | 0.78 | | 2.43 |
| 22 | Indole-3-methyl acetate | C11H11NO2 | [M+H]+ | 189.079 | 189.079 | 1.38 | 9.21 | | 2.27 |
| 23 | Sedoheptulose | C7H14O7 | [M-H]- | 210.074 | 210.074 | -1.11 | 0.63 | | 2.14 |
| 24 | 3-hydroxybutyric acid | C4H8O3 | [M-H]- | 104.046 | 104.047 | -14.07 | 1.12 | | 2.07 |
| 25 | corticosterone | C21H30O4 | [M+H]+ | 346.214 | 346.214 | -0.17 | 11.90 | | 1.98 |
| 26 | Palmitoyl carnitine | C23H45NO4 | [M+H]+ | 399.335 | 399.335 | 1.13 | 16.82 | | 1.95 |
| 27 | DL-carnitine | C7H15NO3 | [M+H]+ | 161.105 | 161.105 | 3.10 | 0.83 | | 1.95 |
| 28 | 3a,6b,7b-trihydroxy-5b-cholic acid | C24H40O5 | [M+CH_2_O_2_-H]- | 454.293 | 454.293 | 0.15 | 15.67 | | 1.86 |
| 29 | Deoxycholic acid | C24H40O4 | [M-H]- | 392.292 | 392.293 | -1.10 | 15.38 | | 1.71 |
| 30 | choline | C5H13NO | [M+H]+ | 103.100 | 103.099 | 8.73 | 0.55 | | 1.61 |
| 31 | 3-dehydrocarnitine | C7H13NO3 | [M+H]+ | 159.090 | 159.090 | -0.21 | 0.77 | | 1.61 |
| 32 | β-guanidino propionic acid | C4H8O3 | [M-H]- | 104.047 | 104.047 | -3.98 | 1.44 | | 1.55 |
| 33 | L-arginine | C6H14N4O2 | [M+H]+ | 174.112 | 174.112 | 0.66 | 0.74 | | 1.53 |
| 34 | D-glucose | C6H12O6 | [M+CH_2_O_2_-H]- | 226.068 | 226.068 | -0.84 | 0.65 | | 1.47 |
| 35 | β-murine cholic acid | C24H40O5 | [M+CH_2_O_2_-H]- | 454.293 | 454.293 | 0.15 | 15.67 | | 1.86 |
| 36 | isoquinoline | C9H7N | [M+H]+ | 129.058 | 129.058 | 3.26 | 9.21 | | 1.35 |
| 37 | Pyridoxamine 5' -phosphate | C8H13N2O5P | [M+H]+ | 248.054 | 248.056 | -10.03 | 3.16 | | 1.25 |
| 38 | N-acetyl leucine | C8H15NO3 | [M+H]+ | 173.105 | 173.105 | 0.90 | 1.05 | | 3.73 |
| 39 | Hippuric acid | C9H9NO3 | [M-H]- | 179.057 | 179.058 | -6.11 | 4.45 | | 1.20 |
| 40 | D-piperaconic acid | C6H11NO2 | [M+H]+ | 129.079 | 129.079 | 0.55 | 0.75 | | 1.19 |
| 41 | Glycocholic acid | C26H43NO6 | [M-H]- | 465.309 | 465.309 | -0.81 | 14.47 | | 1.19 |
| 42 | Phenylbutyric acid | C10H12O2 | [M+H]+ | 164.084 | 164.084 | -0.49 | 11.93 | | 1.17 |
| 43 | 2-hydroxy-3 -(4-3-hydroxyphenyl)  3-acrylate | C9H8O4 | [M+H]+ | 180.042 | 180.042 | -0.05 | 11.57 | | 1.16 |
| 44 | spermidine | C7H19N3 | [M+H]+ | 145.158 | 145.158 | 1.12 | 0.49 | | 1.14 |
| 45 | L-tyrosine | C9H11NO3 | [M+H]+ | 181.074 | 181.074 | 1.03 | 0.85 | | 1.13 |
| 46 | N-methylnicotinamide | C7H8N2O | [M+H]+ | 136.064 | 136.064 | 1.01 | 0.56 | | 1.12 |
| 47 | L-methionine | C5H11NO2S | [M+H]+ | 149.050 | 149.051 | -4.69 | 0.79 | | 1.10 |
| 48 | MG(18:2(9Z,12Z)/0:0/0:0) | C21H38O4 | [M+H]+ | 354.277 | 354.277 | 0.37 | 18.37 | 1.09 | |
| 49 | 4-methylene-L-glutamine | C6H10N2O3 | [M+H]+ | 158.069 | 158.069 | 4.66 | 0.74 | | 1.06 |
| 50 | 2-coumarate | C9H8O3 | [M+H]+ | 164.048 | 164.047 | 1.32 | 0.83 | | 1.06 |
| 51 | LysoPA(18:0/0:0) | C21H42O4 | [M+H]+ | 358.308 | 358.308 | 0.08 | 18.92 | | 1.05 |
| 52 | cycloheximide | C12H23N | [M+H]+ | 181.183 | 181.183 | 2.15 | 7.21 | | 1.03 |
| 53 | D-malic acid | C4H6O5 | [M-H]- | 134.020 | 134.022 | -10.47 | 0.80 | | 1.03 |
| 54 | Glycerophosphocholine | C8H20NO6P | [M+H]+ | 257.103 | 257.103 | 0.76 | 0.54 | | 1.00 |

According to the VIP value in the OPLS-DA model, the screening conditions were as follows: VIP > 1, *P* < 0.05. A total of 54 potential biomarkers with significant differences between the model group and the blank group were screened.
