# Supplementary material for: Study on semi-bionic extraction of Astragalus polysaccharide and its anti-aging activity in vivo
Source: Front Nutr. 2023 Jul 17;10:1201919. doi: 10.3389/fnut.2023.1201919 (PMC10389262; doi:10.3389/fnut.2023.1201919)
Supplement: Supplementary file 3 [file Table_3.docx]

**Table S3.** Differential metabolites of rat serum in the model group and administration group

| **Number** | **metabolite** | **molecular formula** | **Parent ion** | **m/z** | | **Deviation**  **/ppm** | **retention time** | **VIP** |
| --- | --- | --- | --- | --- | --- | --- | --- | --- |
|  |  |  |  | **measured value** | **predicted value** |  | **/min** |  |
| 1 | Indole acrylic acid | C11H9NO2 | [M+H]+ | 187.063 | 187.063 | 0.86 | 2.94 | 7.49 |
| 2 | Phytosphingosine | C18H39NO3 | [M+H]+ | 317.293 | 317.293 | -0.20 | 13.00 | 6.32 |
| 3 | D-tryptophan | C11H12N2O2 | [M+H]+ | 204.090 | 204.090 | 0.40 | 3.19 | 4.33 |
| 4 | valine | C5H11NO2 | [M+H]+ | 117.079 | 117.079 | 1.81 | 0.74 | 4.49 |
| 5 | Monoethylhexyl  phthalic acid | C16H22O4 | [M+H]+ | 278.152 | 278.152 | 0.07 | 11.93 | 3.06 |
| 6 | Larch resin alcohol | C20H24O6 | [M-H]- | 360.157 | 360.157 | -0.72 | 13.13 | 2.93 |
| 7 | L-ortholeucine | C6H13NO2 | [M+H]+ | 131.095 | 131.095 | 0.62 | 1.21 | 3.40 |
| 8 | corticosterone | C21H30O4 | [M+H]+ | 346.214 | 346.214 | -0.17 | 11.90 | 1.98 |
| 9 | Nicotinamide | C6H6N2O | [M+H]+ | 122.048 | 122.048 | 1.86 | 0.77 | 3.97 |
| 10 | choline | C5H13NO | [M+H]+ | 103.100 | 103.099 | 8.73 | 0.55 | 1.61 |
| 11 | Leucine | C6H13NO2 | [M+H]+ | 131.095 | 131.095 | 0.93 | 0.97 | 4.12 |
| 12 | creatine | C4H9N3O2 | [M+H]+ | 131.070 | 131.070 | 2.24 | 0.74 | 3.08 |
| 13 | Indole-3-acetic acid | C11H11NO2 | [M+H]+ | 189.079 | 189.079 | 1.38 | 9.21 | 2.27 |
| 14 | methyl | C_9_H_11_NO_2_ | [M+H]+ | 165.079 | 165.079 | 0.25 | 0.84 | 5.61 |
| 15 | L-phenylalanine | C6H13NO2 | [M+H]+ | 131.094 | 131.095 | -3.88 | 0.85 | 3.03 |
| 16 | isoleucine | C8H20NO6P | [M+H]+ | 257.103 | 257.103 | 0.76 | 0.54 | 1.00 |
| 17 | Glycerophosphocholine | C10H12O2 | [M+H]+ | 164.084 | 164.084 | -0.49 | 11.93 | 1.17 |
| 18 | Phenylbutyric acid | C6H11NO2 | [M+H]+ | 129.079 | 129.079 | 0.55 | 0.75 | 1.19 |
| 19 | D-piperaconic acid | C11H9NO2 | [M+H]+ | 187.063 | 187.063 | 2.94 | 1.03 | 2.23 |
| 20 | Indoleacetic acid | C7H14O7 | [M-H]- | 210.074 | 210.074 | -1.11 | 0.63 | 2.14 |
| 21 | DL-carnitine | C7H15NO3 | [M+H]+ | 161.105 | 161.105 | 3.10 | 0.83 | 1.95 |
| 22 | Acetyl L-carnitine | C9H17NO4 | [M+H]+ | 203.116 | 203.116 | -1.12 | 0.73 | 4.60 |
| 23 | 1-palmitoyl glycerol phosphocholine | C24H50NO7P | [M+H]+ | 495.333 | 495.332 | 0.79 | 18.14 | 2.14 |
| 24 | Hemolytic PC | C26H50NO7P | [M+H]+ | 519.333 | 519.332 | 1.21 | 17.88 | 1.85 |
| 25 | (18:2(9Z,12Z)) | C9H7N | [M+H]+ | 129.058 | 129.058 | 3.26 | 9.21 | 1.35 |
| 26 | isoquinoline | C6H8O7 | [M-H]- | 192.026 | 192.027 | -5.33 | 0.90 | 1.76 |
| 27 | Citric acid | C25H29N3 | [2M+H]+ | 742.472 | 742.471 | 0.40 | 12.76 | 1.73 |
| 28 | Gentian violet | C9H9NO3 | [M-H]- | 179.057 | 179.058 | -6.11 | 4.45 | 1.20 |
| 29 | Hippuric acid | C7H11NO2 | [M+H]+ | 141.079 | 141.079 | 1.50 | 0.78 | 2.79 |
| 30 | L-hydglycine | C7H13NO3 | [M+H]+ | 159.089 | 159.090 | -0.33 | 0.83 | 1.65 |
| 31 | 3-dehydrocarnitine | C8H15NO3 | [M+H]+ | 173.105 | 173.105 | 0.90 | 1.05 | 3.73 |
| 32 | N-acetyl-l - | C7H8N2O2 | [M+H]+ | 152.059 | 152.059 | 0.61 | 0.85 | 1.59 |
| 33 | Leucine | C4H8O3 | [M-H]- | 104.048 | 104.047 | 5.15 | 1.12 | 2.07 |
| 34 | N1-methyl-2-pyridinone-5-formamide | C10H11NO3 | [M+H]+ | 193.074 | 193.074 | 1.43 | 4.79 | 1.76 |
| 35 | 3-hydroxybutyric acid | C5H7NO3 | [M+H]+ | 129.043 | 129.043 | 2.61 | 0.84 | 1.38 |
| 36 | Phenylacetyl glycine | C15H20O5 | [M-H]- | 280.135 | 280.135 | -0.86 | 12.50 | 1.37 |
| 37 | 1-pyrroline-4-hydroxy-2-carboxylate | C11H20N2O3 | [M+H]+ | 228.147 | 228.147 | -0.89 | 0.78 | 2.43 |
| 38 | 8' -hydroxyl shedding material | C9H17NO3 | [M+H]+ | 187.121 | 187.121 | -0.61 | 0.81 | 1.30 |
| 39 | Hydroxyproline - | C4H9N3O2 | [M+H]+ | 131.070 | 131.069 | 4.22 | 1.03 | 1.30 |
| 40 | Leucine | C24H40O4 | [M+H]+ | 392.292 | 392.293 | -1.40 | 17.33 | 1.29 |
| 41 | Dibutyl phthalate | C16H22O4 | [M+H]+ | 278.152 | 278.152 | 0.18 | 15.65 | 1.23 |
| 42 | L-glutamine | C5H10N2O3 | [M+H]+ | 146.069 | 146.069 | 1.01 | 0.74 | 1.20 |
| 43 | Sphingosine | C18H37NO2 | [M+H]+ | 299.283 | 299.282 | 0.50 | 15.53 | 1.20 |
| 44 | Genistein | C15H10O5 | [M-H]- | 270.053 | 270.053 | -0.05 | 10.83 | 1.16 |
| 45 | D-proline | C5H9NO2 | [M+H]+ | 115.064 | 115.063 | 3.66 | 0.77 | 1.09 |
| 46 | Hemolytic PC(18:1(9Z)) | C26H52NO7P | [M+H]+ | 521.349 | 521.348 | 1.25 | 18.29 | 1.09 |
| 47 | Chenodeoxycholic acid | C24H40O4 | [M+CH_2_O_2_-H]- | 438.298 | 438.298 | 0.25 | 17.34 | 1.08 |
| 48 | D-glucose | C6H12O6 | [M+CH_2_O_2_-H]- | 226.068 | 226.068 | -0.84 | 0.65 | 1.47 |

According to the VIP value in the OPLS-DA model, the screening conditions were as follows: VIP > 1, *P* < 0.05. A total of 48 potential biomarkers with significant differences between the model group and the drug group were screened.
